# Advances in Implant-Supported Overdentures: A Review of Recent Attachment Systems

**DOI:** 10.7759/cureus.105520

**Published:** 2026-03-19

**Authors:** Jad Sabbagh, Fabien Fayez Jarjour, Joseph Nassif, Sara Saba, Maha Ghotmi

**Affiliations:** 1 Prosthodontics, Saint Joseph University of Beirut, Beirut, LBN; 2 Faculty of Dentistry, Lebanese University, Beirut, LBN; 3 Prosthodontics, Lebanese University, Beirut, LBN

**Keywords:** attachment system, dental implant, implant-supported overdenture, overdenture, retention

## Abstract

Edentulism continues to present significant challenges in prosthodontic care, with conventional complete dentures (CDs) often failing to provide adequate retention and stability, particularly for the mandible. Implant-supported overdentures (ISODs) have transformed the rehabilitation of edentulous patients by bridging the gap between traditional removable prostheses and fixed implant restorations, offering enhanced stability, retention, and masticatory function while maintaining cost-effectiveness. The McGill consensus established the two-implant mandibular overdenture as the first-choice standard of care for edentulous mandibles. For maxillary cases, anatomical limitations typically necessitate a minimum of four implants. The success of ISOD treatment depends critically on appropriate attachment system selection, considering factors such as available inter-arch space, implant angulation, and maintenance requirements. This article aims to provide a comprehensive review of ISODs, exploring the evolution of attachment systems from early ball and bar designs to contemporary compact systems, including Locator, Locator R-Tx (Zest Dental Solutions, Carlsbad, CA, USA), and OT Equator (Rhein83 S.r.l., Bologna, Italy) attachments. Each system's specific advantages, limitations, space requirements, tolerance to implant divergence, maintenance protocols, and common complications are examined, emphasizing the importance of systematic follow-up care for long-term success.

## Introduction and background

Edentulism, or the complete loss of teeth, remains a widespread, irreversible condition that significantly impacts the quality of life for the aging population. While systemic health improvements have reduced overall rates, recent systematic reviews confirm that the demand for complete-arch rehabilitation remains high [1]. For decades, the conventional complete denture (CD) was considered the gold standard; however, randomized controlled trials have consistently demonstrated its limitations. Specifically, patients often report significant dissatisfaction with mandibular CDs due to a lack of stability and retention, with studies showing that only about 35% of CD wearers are satisfied with their lower prosthesis compared to higher satisfaction rates in the maxilla [2,3].

The transition from conventional dentures to implant-supported protocols is driven by the need to arrest the progressive resorption of the alveolar ridge. Longitudinal evidence suggests that conventional dentures can accelerate residual ridge resorption due to unphysiological pressure on the soft tissues [4-6]. In contrast, the introduction of dental implants provides a masticatory load-sharing mechanism. Systematic reviews and meta-analyses, such as those by Van de Winkel et al. and Sutariya et al., highlight that implant-supported overdentures (ISODs) significantly improve Oral Health-Related Quality of Life (OHRQoL) scores and masticatory efficiency compared to CDs [1,4,7].

Central to the success of an ISOD is the attachment system, which serves as the mechanical interface between the implants and the prosthesis. The choice of attachment, ranging from splinted bar systems to individual attachments, directly dictates the distribution of stress to the peri-implant bone and the retentive longevity of the prosthesis [8,9]. While fixed implant-supported prostheses offer maximum stability, ISODs are often preferred in clinical scenarios involving significant alveolar bone loss, where the denture flange is required to provide lip support and facial aesthetics [7,8].

Despite the clear benefits of ISODs, the ideal attachment system remains a subject of clinical debate. Newer compact individual attachments have been developed to address the spatial limitations and high maintenance requirements of traditional ball and bar designs [1,8]. However, clinicians face challenges regarding component wear, loss of retention, and the need for frequent replacement of retentive inserts [3,5].

The aim of this article is to provide an in-depth analysis of ISODs, tracing the development of attachment systems from traditional ball and bar configurations to modern compact options such as Locator, Locator R-Tx (Zest Dental Solutions, Carlsbad, CA, USA), and OT Equator (Rhein83 S.r.l., Bologna, Italy) designs. The specific benefits, drawbacks, spatial requirements, capacity to accommodate implant angulation, maintenance strategies, and frequently encountered complications are evaluated for each system, highlighting the critical role of structured follow-up protocols in achieving durable clinical outcomes. 

## Review

A comprehensive qualitative literature review was conducted, involving a search for relevant articles in the PubMed/Medical Literature Analysis and Retrieval System Online (MEDLINE), Scopus, and Embase databases. The search utilized the following terms: "attachment system," "dental implant," "implant-supported overdenture," "overdenture," and "retention," combined using the Boolean operators “OR” and “AND.” The period considered for study selection ranged from 2000 to 2025. Inclusion criteria consisted of clinical studies, in vitro trials, case reports, and technical reviews published in English. To ensure clinical relevance, we excluded studies that focused solely on fixed restorations or those with insufficient data on attachment systems. After the removal of duplicates and initial screening, 48 articles were ultimately included in this review. Data were categorized and synthesized based on the type of attachment system, biomechanical classification, minimum vertical space requirements, and maximum divergence compensation limits. Due to the heterogeneity of the included studies and the descriptive nature of the technical specifications provided by manufacturers, a meta-analysis was not performed. Instead, a narrative synthesis was conducted to balance academic findings with practical clinical applications. To minimize bias, each included paper was evaluated based on the appropriateness of the research design and the clinical relevance of its findings. While this review follows the spirit of Preferred Reporting Items for Systematic Reviews and Meta-Analyses (PRISMA) guidelines for transparency, a narrative approach was maintained to allow for a flexible and comprehensive exploration of this evolving field.

Mandibular vs. maxillary ISODs

The challenges associated with mandibular CDs have led to significant advancements in treatment modalities. ISODs have rapidly evolved into a highly refined and predictable treatment option for edentulous mandibles [2,4].

While a minimum of two implants can provide adequate support for mandibular overdentures, using more than two implants offers added protection against potential implant failure over the patient’s lifetime. However, there is an inverse relationship between the number of implants and the cost-effectiveness of treatment. To balance these factors, a group of scientists and expert clinicians convened at McGill University in Montreal, Canada, to develop a consensus statement on the efficacy of overdentures for edentulous patients [6]. Their recommendation established the mandibular two-implant overdenture as the first choice in treating edentulous mandibles, due to its optimal balance of effectiveness and cost-efficiency [6,10].

For the maxillary arch, treatment planning considerations differ significantly. Conventional CDs can often provide satisfactory outcomes in the maxilla due to enhanced retention and stability from palatal coverage; however, certain cases may require implant support. When implant treatment is indicated for the maxillary arch, a minimum of four implants is typically recommended to achieve adequate support and stability for an ISOD. According to Branemark et al. [9], four well-positioned implants are sufficient to support an overdenture. Therefore, given this minimum requirement of four implants, it becomes more cost-effective to place two additional implants and opt for a fixed prosthesis, which offers superior functional and biomechanical advantages. This treatment approach maximizes the return on investment for the patient while providing optimal clinical outcomes [2,9,11].

Advantages and challenges of ISOD

Advantages

Multiple studies, including randomized controlled trials with short- and long-term follow-ups, have demonstrated the clinical superiority of ISODs over conventional dentures [6,11,12]. In the mandible, ISODs supported by two implants demonstrate high predictability, with success rates often exceeding 95% [6]. Biologically, these systems provide a significant advantage by limiting residual ridge resorption, which can reach several millimeters per year with conventional dentures but is stabilized by the functional loading provided by implants [6,11]. This is further supported by Reddy et al., who demonstrated that the height of the posterior ridge can actually increase over time due to Wolff’s Law, where functional mechanical loading stimulates bone apposition and reverses disuse atrophy [13].

Functionally, as few as two to four implants provide the necessary stability and retention to significantly improve masticatory efficiency and reduce soft tissue irritation caused by prosthesis movement [11]. Furthermore, patient-based outcomes indicate that ISODs are rated significantly higher than traditional dentures, with clinically relevant improvements in OHRQoL [12]. Specifically, patients report marked reductions in functional limitations, psychological discomfort, and physical pain [12]. These positive outcomes persist regardless of demographic characteristics or specific prosthetic designs, making ISODs a highly effective and cost-efficient standard of care for the edentulous arch [6,11,12].

Challenges

While ISODs offer significant advantages over conventional dentures, they also come with their own set of challenges. A primary concern is the necessity for implant surgery, which can involve multiple procedures, particularly if bone grafting is needed. This surgical aspect introduces additional risks and recovery time compared to conventional dentures [14].

The maxilla poses particular difficulties in ISOD treatment due to its inherently lower bone quality, often requiring a greater number of implants to adequately support a prosthesis. A significant obstacle that practitioners frequently encounter is the severe resorption of alveolar bone in the posterior maxilla. This anatomical limitation can complicate optimal implant placement in terms of number, distribution, and length, potentially contributing to higher failure rates compared to mandibular ISODs. Survival rates for maxillary ISODs vary widely in the literature, ranging from 72.4% to 100%. This variability underscores the critical importance of meticulous treatment planning, careful case selection, and skilled execution to maximize the success of maxillary ISODs [14].

Patients opting for ISODs must be prepared for potential complications, including peri-implantitis (an inflammation affecting the tissues surrounding implants leading to bone destruction) and implant failure, which can occur at any stage, immediately post-implantation or later. These complications are not unique to ISODs but are risks associated with all dental implants that must be vigilantly prevented through proper oral hygiene and regular professional monitoring [14].

ISODs demand a higher level of ongoing maintenance and preventive care compared to conventional dentures. Patients must commit to diligent oral hygiene practices and regular professional check-ups, which can be particularly challenging for those who have historically struggled to maintain their natural teeth [6,14]. This intensified maintenance schedule is crucial for ensuring the longevity of both the implants and the attachment systems, maintaining optimal peri-implant health, preserving denture retention, and extending the overall lifespan of the prosthesis [14].

Attachment systems

The clinical success of an ISOD is governed by the attachment system, which acts as the mechanical interface between the implants and the prosthesis. An attachment system is a mechanical component consisting of an abutment connected to the implant and a housing with a retentive insert integrated into the prosthesis [8,15].

These systems are classified based on configuration as either splinted or individual. Splinted attachments involve connecting implants via a bar structure to provide cross-arch stabilization, while individual attachments allow implants to function as independent units, facilitating easier hygiene access [15]. Furthermore, they are categorized by their functional characteristics into rigid and resilient attachments. Rigid attachments, primarily bar systems, prevent movement between components during function [14,15]. In contrast, resilient attachments, such as ball, magnets, Locator, and OT Equator, permit a controlled degree of movement to distribute functional forces and protect the peri-implant bone [14,15].

The selection of these systems is a biomechanical necessity dictated by ridge anatomy, inter-arch space, and implant angulation [16]. The morphology of the residual ridge directly influences this selection; in cases of advanced resorption where the ridge is narrow or significantly atrophied (Atwood Class IV or V), splinted bar systems are often indicated to provide necessary cross-arch stabilization and prevent prosthesis rotation [16]. Conversely, in wider ridges with adequate bone volume and height, individual stud attachments may be utilized to simplify hygiene maintenance and reduce technical complexity. Furthermore, the thickness of the overlying mucosa is a critical determinant; a minimum of 2-3 mm of soft tissue is typically required to allow for a proper emergence profile of the abutment and to ensure that the attachment housing does not cause chronic gingival irritation or inflammatory hyperplasia [15,16].

Adequate inter-arch space (defined as the vertical distance between the opposing dental arches) is the most important factor in prosthetic design, as insufficient space leads to thin, fracture-prone acrylic bases or premature wear of the retentive components [17,18]. Evaluation must occur prior to implant surgery using diagnostic wax-ups or 3D planning software to ensure specific minimum requirements are met. Among the various options, bar attachments demand the greatest vertical height, typically requiring 13-14 mm of space to accommodate the bar, clip mechanism, and prosthetic materials [8,11,14]. Ball attachments generally require 10-12 mm of vertical space, while compact studs such as Locator or OT Equator are the most space-efficient, requiring only 7-9 mm of vertical space [8,14]. Proper assessment involves measuring the distance from the implant platform to the incisal edge or occlusal plane. Failure to provide this space necessitates either surgical bone reduction (alveoloplasty) or the selection of a more compact system to avoid prosthetic failure [17,19,20].

The capacity of a system to compensate for implant divergence is vital for long-term success. While traditional ball attachments can generally accommodate up to 10-15° of divergence per implant, significant challenges arise when implants are non-parallel [8,16,21]. Modern systems have expanded these limits; for instance, the Locator R-Tx and OT Equator are designed to manage 50-60° of total divergence between two implants [1,8,22]. Exceeding these clinical limits results in accelerated one-sided wear of the retentive inserts, loss of retention, and increased lateral stress on the peri-implant bone. Finite element analysis (FEA) by Khurana et al. confirms that as divergence increases beyond the system's intended capacity, stress concentration at the implant-bone interface rises exponentially, which can lead to marginal bone loss or abutment screw loosening [16,23,24].

Table 1 summarizes the primary attachment systems based on the biomechanical and clinical criteria discussed:

**Table 1:** Summary of biomechanical classifications, vertical space requirements, and divergence limits for common overdenture attachment systems. Locator/Locator R-Tx: Zest Dental Solutions, Carlsbad, CA, USA; OT Equator: Rhein83 S.r.l., Bologna, Italy

Attachment System	Inter-arch Space Required	Max Total Divergence	Biomechanical Type	Main Advantage
Bar Attachment	13 - 14 mm	N/A (Parallelism Required)	Rigid / Splinted	Maximum stability; cross-arch stabilization
Ball Attachment	10 - 12 mm	10° - 15°	Resilient / Individual	Cost-effective; historical reliability
Magnetic Attachment	9 - 10 mm	Unlimited	Non-Resilient	Ease of use for limited dexterity
Locator	8 - 9 mm	40°	Resilient / Individual	Dual retention
OT Equator	7 - 8 mm	50°	Resilient / Individual	Smallest vertical profile available
Locator R-Tx	8 - 9 mm	60°	Resilient / Individual	Highest divergence compensation

In the following sections, we will explore the historical development and clinical applications of various attachment systems, from the earliest attachment systems to the most advanced designs, examining their specific advantages, limitations, ideal clinical scenarios, and the key considerations that guide clinicians in choosing the most suitable attachment system for each individual case.

Ball Attachments

**Figure 1:**
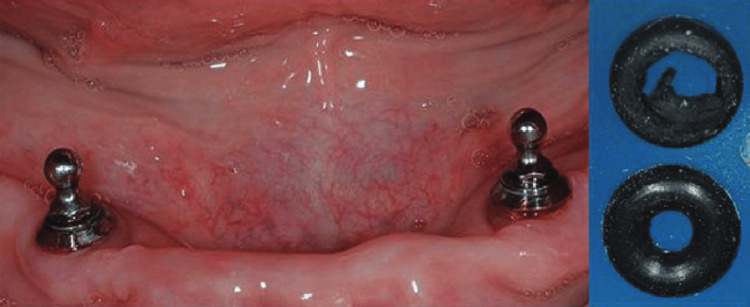
Clinical view of ball attachment abutments (patrices) and their corresponding prosthetic housings (matrices). The image is reproduced with permission from John Wiley and Sons [20].

Ball attachments, introduced in the 1960s, marked the beginning of ISOD systems and represented one of the earliest attempts to improve prosthetic retention and stability. The system's straightforward design, consisting of a spherical male component (patrix) attached to the implant and a corresponding female component (matrix) housed within the denture base (Figure 1), offered clinicians their first reliable method for securing ISODs, and it continues to serve as a reliable option in suitable cases. The attachment relies on mechanical retention through an O-ring or plastic cap matrix that engages and grips the undercuts of the ball abutment, providing both retention and some degree of resilience [8,20].

Ball attachments require a minimum inter-arch space of 10-12 mm to accommodate the ball patrix height (3-4 mm), matrix space (2-3 mm), and denture base with acrylic teeth (4-5 mm), resulting in a higher vertical profile compared to newer attachment systems. These systems demonstrate optimal performance with parallel or minimally divergent implants, tolerating a maximum divergence of approximately 10-15 degrees between implants, as greater angulation can lead to accelerated component wear [21,22].

Clinical evidence supports the high predictability of this system. Longitudinal studies have reported a five-year implant survival rate of 100% when using ball attachments for mandibular two-implant overdentures [22]. When compared to other systems, such as Locator attachments, ball attachments demonstrate comparable one-year success rates, with no significant differences in marginal bone loss or peri-implant soft tissue health [20,23]. However, some retrospective data suggest that while implant survival is high, the ball system may require more frequent prosthetic maintenance over time [21].

Patient-reported outcomes for ball attachments are generally positive. Research indicates significant improvements in patient comfort, chewing ability, and overall satisfaction compared to conventional dentures [20]. While some studies suggest that Locator attachments might offer slightly higher satisfaction scores in the short term, five-year follow-ups show no statistically significant difference in OHRQoL or general satisfaction between the two systems [22,23].

From an operator perspective, ball attachments are recognized for their clinical simplicity and cost-effectiveness [22]. However, they are associated with specific complications, most notably the loss of retention due to matrix wear and the need for frequent replacement of retentive components [14,21]. Comparative systematic reviews indicate that ball attachments often exhibit higher rates of "technical complications," such as the need for matrix activation or replacement, compared to bar or Locator systems [23]. Despite these maintenance requirements, the procedure for component replacement remains straightforward and minimally invasive for the operator [8,22].

Bar Attachments

**Figure 2:**
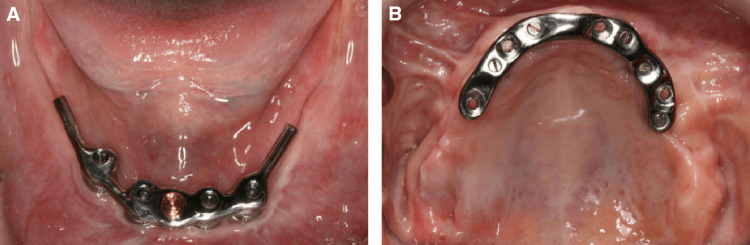
Clinical view of bar-supported overdenture frameworks. A: metal bar supported by four mandibular implants; B: metal bar supported by five maxillary implants. The image is reproduced with permission from John Wiley and Sons [11].

The bar attachment system, introduced shortly after ball attachments in the late 1960s, represented a significant advancement in implant overdenture technology. This system, particularly popularized by Dr. Eugen Dolder, introduced the concept of splinting implants to provide improved stability and force distribution. Bar attachments consist of a metal bar connecting two or more implants (Figure 2) with a corresponding clip or rider integrated into the denture base [8,14].

Regarding fabrication methods, bars can be produced using several techniques that have evolved significantly over time [8,24]. Traditional casting with noble alloys, particularly gold, includes systems like Dolder® (Cendres + Métaux SA, Biel/Bienne, Switzerland) but was prone to inaccuracies due to metal contraction during cooling. Pre-milled castable plastic patterns can be used for various bar designs, with the Hader® system (Sterngold Dental, LLC, Attleboro, MA, USA) being a notable example. Modern computer-aided design and manufacturing (CAD/CAM) techniques have greatly improved precision and reduced complications associated with traditional casting methods. This process involves optically scanning stone casts and wax setups to generate 3D digital models for milling, using titanium or titanium alloy blocks [8,24].

Bar attachments are available in various cross-sectional profiles, including round "Hader," egg-shaped, and parallel-sided U-shaped "Dolder" designs. These systems achieve optimal outcomes when implants are placed parallel to each other at an inter-implant distance of 20-22 mm [25,26].

Clinical studies highlight the high predictability and mechanical robustness of bar systems. In the maxilla, a case series by Tallarico et al. demonstrated a 100% implant and prosthesis survival rate over the short term, with significant preservation of peri-implant bone [27]. Comparative evaluations indicate that bar attachments provide superior stress distribution and cross-arch stabilization compared to individual axial attachments, particularly in cases with limited bone volume [16,26]. Specifically, a three-dimensional FEA by Khurana et al. demonstrated that bar systems effectively distribute masticatory loads, resulting in lower stress concentration on individual implants compared to ball systems, though the height of the bar remains a critical factor in stress levels [24].

Regarding patient outcomes, bar-retained overdentures are associated with high levels of patient comfort and satisfaction. Research by Mañes Ferrer et al. comparing bar systems to individual attachments in the maxilla found that bar-retained prostheses offered higher mechanical stability and required fewer interventions for loss of retention, contributing to improved long-term patient confidence [26,27].

However, bar systems are associated with specific operator-reported complications and strict design requirements. Bar systems demand the most vertical space among all attachment systems, requiring a minimum of 13-14 mm to accommodate the bar height (3-4 mm), clip mechanism (2-3 mm), denture base and acrylic teeth (6-7 mm), and necessary clearance for hygiene maintenance under the bar (2-3 mm) [25,27]. A decisive limitation of this configuration is its inability to tolerate divergent implants; for a bar system to function effectively without mechanical strain, the implants must be placed as parallel as possible to ensure a passive fit of the substructure and a predictable path of insertion for the overdenture [16,25]. While they require less frequent maintenance of the retentive clips compared to the frequent matrix replacements needed for ball systems, they are prone to mechanical issues such as abutment screw loosening and a higher risk of denture base fractures due to the significant space required for the bar housing [16,26]. Furthermore, the complexity of the design can lead to biological complications, such as gingival hyperplasia, if the patient cannot maintain adequate hygiene in the clearance space beneath the bar [16,27].

Magnetic Attachments

Magnetic attachments were introduced in the 1970s, offering a fundamentally different approach to overdenture retention. Initially, these systems employed opposing magnets embedded in the maxillary and mandibular denture bases, using repulsive forces to maintain the dentures in place. However, this early approach provided unreliable and weak retention, with the magnets prone to corrosion [28].

Modern magnetic attachment systems have significantly improved upon this design. They typically consist of a keeper (an abutment attached to the implant via a screw) and a magnet fixed to the denture's fitting surface. To prevent corrosion and maintain attractive force, the magnets are usually encased in a corrosion-resistant sealant. These current designs offer enhanced stability and stronger attractive forces compared to the earlier versions [8,29].

Magnetic attachments are particularly beneficial for patients with reduced manual dexterity who may struggle with other retention systems. They provide a viable solution for those who find other systems challenging to insert or remove. Unlike bars, balls, or Locators, magnet-retained dentures possess a degree of 'self-location' over short distances, reducing the need for precise placement by the patient. Their space requirements are minimal, needing only 9-10 mm of inter-arch space, which includes the keeper height (2-3 mm), magnet housing (2-3 mm), and the required space for the denture base and acrylic teeth (4-5 mm). This compact design makes them suitable for cases with limited inter-arch space, and their simple configuration, with minimal undercuts, facilitates easier cleaning [29,30].

One significant advantage of magnetic systems is their ability to maintain an attractive force regardless of implant divergence, allowing for effective retention even when implants are not parallel. Another benefit is that these attachments minimize the transfer of horizontal stress to the implants and surrounding bone during denture insertion and removal, potentially contributing to long-term implant health [28,30].

Nevertheless, their use in clinical practice remains limited. This is primarily because magnetic attachments are generally considered the least retentive among all available overdenture attachment systems. This significant limitation, coupled with the tendency for their retention force to diminish over time, often results in more frequent maintenance requirements. With the advent of more reliable attachment systems like Locators and OT Equators that offer superior retention and durability, magnetic attachments have largely fallen out of favor in contemporary clinical practice [8,28,30].

Locator Attachments

**Figure 3:**
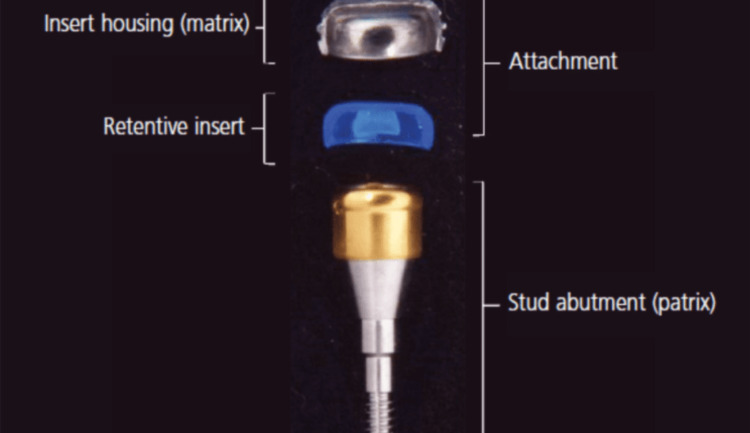
Schematic representation of the Locator attachment system, showing the abutment, retentive insert, and metal cap. The image is reproduced from an open-access article [19].

The Locator attachment system, introduced by Zest Anchors in 2001, marked a revolutionary advancement in overdenture attachment technology. This system rapidly gained widespread acceptance due to its innovative features addressing many limitations of earlier systems [21]. The Locator system consists of two primary components working in harmony: the abutment (female component) that attaches directly to the implant and features precisely engineered retention surfaces both internally and externally, and the male component comprising a replaceable nylon insert housed within a durable titanium alloy cap that secures into the denture base (Figure 3) [14,24].

This design enables a unique dual retention mechanism that fundamentally distinguishes the Locator system from conventional attachments [8,31]. The external retention mechanism utilizes a shallow undercut around the abutment periphery, providing complete external margin attachment that engages simultaneously with the outer surface of the abutment, creates primary stability through mechanical retention, and distributes forces over a broader surface area [14,24]. The internal retention mechanism employs a slightly oversized nylon male component that creates secure press-fit engagement inside the inner metal ring, provides additional mechanical and frictional retention, functions independently of external retention, and allows for customization of retention forces [14,24]. This configuration allows the patrix, when fully inserted, to engage both the inner and outer surfaces of the matrix, hence the term "dual retention." The dual retention design effectively doubles the available retention surface compared to traditional systems, resulting in enhanced prosthesis stability during function, more predictable and consistent retention levels, improved distribution of masticatory forces, and extended component longevity with reduced maintenance requirements [8,14].

One of the system's most notable features is its compact design and low vertical profile, making it suitable for cases with limited inter-arch space. The Locator attachment itself has a minimal footprint, measuring only 3.77 mm in height and 5 mm in width. When considering the complete prosthetic setup, the system requires a minimum total vertical space of 8.5 mm. This total space accommodates the female abutment with minimal tissue exposure (1.5 mm), the metal housing with a nylon male (2.27 mm), and the required space for the denture base and acrylic teeth (4-5 mm). Female abutments are available in different gingival heights ranging from 1 mm to 6 mm to accommodate different implant placement depths [32].

The system demonstrates remarkable adaptability to non-parallel implants, with standard inserts accommodating up to 20° between implants and extended range options managing up to 40° total divergence. The Locator male insert features an innovative pivoting design within its metal housing that allows the insert to pivot during insertion, removal, and mastication. This pivoting action enables the standard inserts to accommodate up to 10° in each direction, providing a total range of 20° between implants, while extended range inserts allow up to 20° of divergence per implant, accommodating a total of 40° between implants [14,32].

The Locator system offers multiple retention levels through color-coded nylon inserts for different clinical situations. Standard range inserts, designed for divergence up to 10° per implant or 20° between implants, include clear inserts providing strong retention (5 lbs) for optimal stability, pink inserts offering medium retention (3 lbs) for balanced function, and blue inserts delivering light retention (1.5 lbs) for easier handling (33). Extended range inserts, accommodating divergence up to 20° per implant or 40° between implants, include green inserts for strong retention (3 lbs), orange for medium retention (2 lbs), red for light retention (1.5 lbs), and gray for zero retention (0 lbs), providing a solution for reducing denture retention when desired [32]. 

One common challenge with overdenture attachments is the potential for component damage during insertion if patients fail to properly align the male and female parts. This frequently occurs when patients attempt to seat their overdenture by biting it into place at an incorrect angle, leading to distortion of the attachment components and eventual need for replacement. The Locator attachment system addresses this issue through its innovative self-aligning design. Its unique configuration features rounded contours on the female component that work in conjunction with the nylon male's extended skirt, creating a self-guiding mechanism similar to the guide planes found in partial dentures. This design not only facilitates proper seating of the prosthesis but also reduces component wear, ultimately extending the lifespan of the attachment system and improving patient satisfaction [14,32].

An important element in attachment design is resiliency. This characteristic allows for controlled movement between the implants and the prosthesis, effectively distributing masticatory forces between the implants and the tissue-bearing areas. This is achieved through its pivoting design that allows both rotational and vertical movement. Through this design, stress transfer to the supporting implants is minimized while accommodating natural tissue movement during function [8,14].

Successful long-term outcomes with Locator attachments require regular monitoring of nylon insert wear patterns, periodic replacement of retentive elements as needed, with most maintenance performed chairside during annual follow-up appointments, and patient education remaining crucial for sustained clinical success [8,14].

OT Equator Attachments

As implant overdenture therapy continued to evolve, the need for a more versatile attachment system became apparent, especially for cases with anatomical limitations. The OT Equator system (Rhein'83), introduced in 2007, addressed this need through its ultra-compact design and enhanced adaptability to divergent implants. The system's reduced dimensions and advanced retention capabilities enabled clinicians to successfully treat cases where space limitations and implant angulation had previously posed significant barriers [33,34].

**Figure 4:**
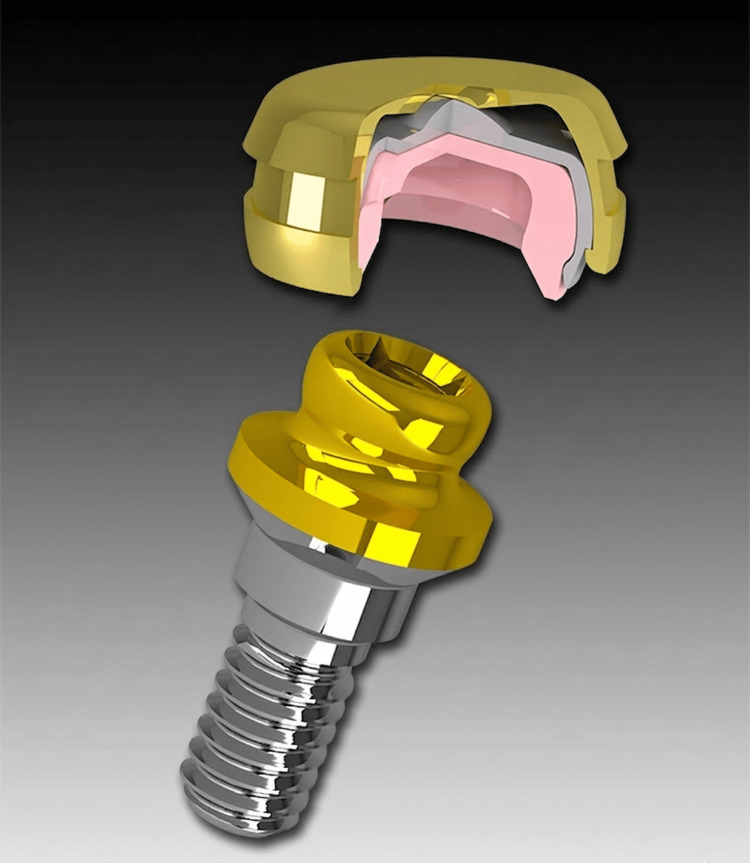
Schematic illustration of the OT Equator attachment system. Image courtesy of Rhein83, Bologna, Italy (www.rhein83.com); permission has been obtained for use in this review.

The OT Equator system (Figure 4) is characterized by its ultra-compact dimensions, representing the smallest attachment system available in the market. With a total vertical height of only 2.1 millimeters and a maximum diameter of 4.4 millimeters, it offers an even lower profile than the Locator system, which measures 3.77mm in height and 5mm in width [33,34].

Regarding the complete prosthetic setup, the system requires a minimum total inter-arch space of 7-7.5 mm. This total space accommodates the female abutment (1.2 mm), the metal housing with retentive insert (0.9 mm), and the required space for the denture base and acrylic teeth (4-5 mm) [34,35]. The male component features a titanium nitride (TiN) coating for enhanced durability, while the female component comprises a stainless-steel housing that accommodates various color-coded retentive caps. The female abutments are available in various gingival heights, ranging from 0.5 mm to 7.0 mm. The system offers four distinct retention levels through differently colored inserts: extra-soft retention (yellow), soft retention (pink), standard retention (clear), and extra-strong retention (green). This range of retention options allows clinicians to customize the retentive force based on clinical requirements [34,35].

**Figure 5:**
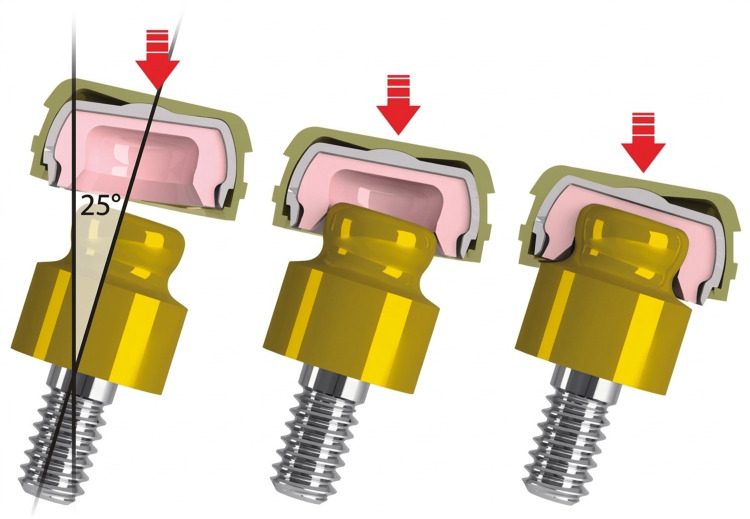
Schematic illustration of the OT Equator Smart Box design, showing the tilting mechanism that enables passive insertion and compensates for up to 50 degrees of total implant divergence. Image courtesy of Rhein83, Bologna, Italy (www.rhein83.com); permission has been obtained for use in this review.

Perhaps the most innovative aspect of the OT Equator system is its Smart Box design (Rhein83 S.r.l., Bologna, Italy) [34]. This technology incorporates a tilting mechanism with a rotating fulcrum that enables passive insertion even in cases with significant implant divergence: up to 50 degrees (Figure 5). This capability surpasses the angular tolerance of traditional attachment systems, including the Locator system, which typically accommodates up to 40 degrees of divergence with extended-range attachments [34,35].

When comparing the OT Equator with the Locator system, several key differences emerge. In a comprehensive in vitro study, Mínguez-Tomás et al. evaluated the retention characteristics of both systems over 14,600 insertion-removal cycles, designed to simulate 10 years of clinical use based on an average of four daily prosthesis removals [35]. The study utilized 10 specimens of each attachment system, with pink nylon inserts (Locator: 13.33 N; Equator: 11.76 N rated retention) mounted on identical implant replicas. Testing revealed that both systems initially demonstrated comparable retention forces (OT Equator: 16.36 ± 2.94 N; Locator: 17.02 ± 2.69 N) and showed an interesting pattern where retention actually increased during the first 1,000 cycles (approximately eight months of simulated use), with the Locator reaching 21.72 ± 7.65 N and the OT Equator reaching 20.16 ± 4.75 N. After this initial period, both systems began to lose retention progressively, with significant differences emerging after 7,500 cycles (equivalent to five years of use), where the Locator system maintained 69.33% of its baseline retention compared to the OT Equator's 45.96%. By the end of the testing period (14,600 cycles), the Locator system retained 49.76% of its initial retention (8.47 ± 2.91 N), while the OT Equator maintained 30.26% (4.95 ± 1.98 N). Despite these differences in retention loss, both systems maintained clinically acceptable retention values throughout the testing period, as the literature suggests that minimum retention forces between 3-8 N are sufficient for adequate overdenture stability [35]. This comparative analysis suggests that while both attachment systems demonstrate adequate long-term clinical performance, the Locator system exhibits superior retention maintenance over time, retaining nearly 50% of its initial retention force after simulated long-term use compared to approximately 30% for the OT Equator system, though both remained within clinically acceptable retention ranges [35].

The clinical advantages of the OT Equator system are particularly evident in cases with severely limited inter-arch space. The reduced vertical profile not only facilitates prosthesis construction but also minimizes the need for excessive prosthetic bulk, ultimately allowing for better aesthetics in the final restoration [36]. The system's simplified component architecture, combined with the Smart Box technology, provides enhanced passive fit and potentially reduced maintenance requirements compared to traditional attachment systems [33,34].

Additionally, the inter-implant distance appears to significantly influence attachment wear, retention, and peri-implant stress distribution. El Mekawy et al. compared wear patterns between 19 mm and 25 mm inter-implant distances for both Locator and OT Equator attachments, finding that the 25 mm distance led to greater surface deformities, micro-voids, and particle loss [37]. These results align with the FEA by Hong et al., which demonstrated that placing implants more anteriorly (corresponding to a 19 mm distance) reduces peri-implant bone stresses, thereby minimizing the wear of the retentive male inserts [38].

However, the selection of inter-implant distance requires a careful biomechanical balance. While a shorter distance (19 mm) offers mechanical advantages for overdenture support and component longevity [21,37], it may increase the risk of prosthesis instability. This is in contrast to the findings of Marin et al., who reported that placing implants too far mesially (e.g., in the lateral incisor area) can result in occlusal complications and unfavorable tipping of the prosthesis during functional loading [39]. Consequently, while a 19 mm distance may preserve attachment integrity, clinicians must ensure the implants are positioned widely enough to provide a stable base and prevent the "seesaw" effect often associated with narrow-base support.

The biomechanical advantages of the OT Equator system extend beyond its compact dimensions. The Smart Box mechanism, with its innovative tilting design and rotating fulcrum, contributes significantly to favorable force distribution across the supporting structures. The elastic material of the retentive matrix allows for the distribution of retentive forces over a larger surface area, contrasting with the Locator's matrices, which concentrate forces in a smaller area. This fundamental design difference results in more sustained retention due to reduced wear at the circumference and reduced stress on the supporting implants [40]. Furthermore, Ameen et al. [41], examining microstrain patterns around implants, have revealed that OT Equator attachments demonstrate more favorable stress distribution patterns, particularly during unilateral and bilateral loading conditions.

The OT Equator's simplified architecture contributes to optimized maintenance protocols, showing notable resilience in maintaining functional retention despite dimensional constraints. In a comprehensive multicenter retrospective analysis by Tallarico et al. involving 194 patients over periods ranging from one to 17 years, the OT Equator demonstrated significantly superior maintenance outcomes compared to Locator attachments [36]. At five-year follow-up examinations, the OT Equator system exhibited better prosthetic survival rates; none of the 89 OT Equator cases experienced prosthetic failure, while four failures occurred among the 39 Locator cases (P = 0.0069). Similarly, mechanical complications were less frequent with the OT Equator system, with only six complications reported in 83 cases compared to 10 complications in 33 Locator cases (P = 0.0289). The authors attributed this superior performance to the OT Equator's elastic retentive matrix material, which appears to better distribute occlusal forces and reduce component wear compared to Locator. Additionally, biological parameters remained stable, with marginal bone loss measurements showing no significant differences between attachment types at one-year (OT Equator: 0.32 mm; Locator: 0.29 mm) and two-year follow-ups (OT Equator: 0.36 mm; Locator: 0.36 mm). When complications did occur, they were predominantly minor technical issues that could be resolved chairside in less than 60 minutes, most commonly involving matrix replacement or retention adjustments [36].

From a biological perspective, Gandhi et al. highlighted that the system's design specifically promotes peri-implant tissue health by maintaining fibromucosal adherence and supporting the formation of a protective gingival barrier, which may contribute to the reduced incidence of inflammation and peri-implantitis observed in clinical practice [42].

In terms of prosthetic workflow, the OT Equator system demonstrates excellent compatibility with both traditional and digital protocols. Tasopoulos et al. [33] and Meneghetti et al. [43] have shown that the system's predictable behavior and standardized components facilitate efficient clinical procedures, particularly in digital workflows incorporating CAD/CAM technology. This adaptability to modern digital dentistry, while maintaining compatibility with conventional techniques, makes it a versatile option in contemporary implant prosthodontics.

Cost-effectiveness analyses and long-term outcome studies further support the system's value in contemporary implant prosthodontics. While initial costs may be comparable to alternative systems, the reduced maintenance requirements and lower complication rates suggest favorable long-term economic benefits for both practitioners and patients [36].

Clinical recommendations for optimal outcomes with the OT Equator system emphasize the importance of careful case selection and thorough treatment planning [34]. Cases with severe space limitations, implant divergence beyond 40 degrees, or situations requiring simplified maintenance protocols may benefit most from this system. However, the choice between attachment systems should always be based on a comprehensive evaluation of individual case requirements, considering factors such as available inter-arch space, implant angulations, anticipated maintenance requirements, and patient-specific factors. While both OT Equator and Locator systems demonstrate clinical efficacy, their distinct characteristics make them suitable for different clinical scenarios. Regular monitoring of retention and wear patterns, combined with appropriate maintenance protocols, remains essential for long-term success regardless of the system selected [34,35].

Both the Locator and OT Equator systems represent significant advancements in attachment technology for ISODs, each offering unique advantages for different clinical scenarios. The Locator system provides excellent long-term retention stability and durability with its dual retention mechanism, while the OT Equator system excels with its ultra-compact design and enhanced tolerance to implant angulation and superior long-term outcomes in terms of prosthetic survival and complications.

Locator R-Tx Attachments

Building upon the success of the original Locator system, Zest Anchors introduced the next-generation Locator R-Tx attachment system in 2016, incorporating several technological advancements while maintaining the familiar clinical protocols [31]. The system introduces DuraTec (Locator R-Tx coating), a proprietary coating combining multiple layers of titanium carbon nitride and TiN, which demonstrates superior mechanical properties compared to the traditional Locator TiN coating, including 30% increased hardness, 25% greater wear resistance, and 65% reduction in surface roughness. The housing's pink anodization improves aesthetics in areas of thin denture acrylic, addressing a common aesthetic concern with previous attachment systems [31,32].

A key innovation in the R-Tx system is the redesigned engagement mechanism, where the nylon retention inserts now engage dual retentive surfaces exclusively on the exterior of the abutment, eliminating internal engagement. By moving all retention to the external surfaces, the design prevents food debris and plaque that might collect in the abutment's central cavity from affecting proper seating [31,32].

The Locator R-Tx system's most significant advancement is its remarkable ability to accommodate up to 60 degrees of divergence between implants, a 50% increase from the original Locator's 40-degree limit [32]. This exceptional angular tolerance represents a breakthrough in attachment technology. The magic of Locator has been that the nylon retention inserts pivot within the denture attachment housing during insertion or removal, resulting in a resilient system that allows patients to seat their overdenture without damaging the components. The Locator R-Tx pivoting technology builds upon this foundation while significantly improving the angular range. Once the retention insert is fully seated on the abutment, the denture attachment housings are able to pivot over the retention insert during masticatory function, giving the system a level of resilience not seen in other attachment systems [31,32].

The denture attachment housing has been redesigned with horizontal grooves and flats that resist vertical and rotational movement. An internal channel at the top of the housing enhances the pivot range of motion [31,32].

The R-Tx system employs a simplified retention insert system with four straightforward retention values: zero, low, medium, and high. This practical approach allows clinicians to easily select and adjust retention levels based on individual patient needs. The improved insert design also demonstrates better resistance to edge deformation, extending component longevity [31,32].

Maintenance and complications

ISODs require systematic maintenance protocols to ensure long-term success and patient satisfaction. Both biological and mechanical aspects must be monitored regularly, as complications can significantly impact prosthetic function and patient quality of life [44,45]. 

Biological Complications

The maintenance of peri-implant health requires careful attention to several parameters through regular professional monitoring. Clinicians should perform systematic evaluations of peri-implant pocket depths and assess bleeding on probing during recall appointments. Detection of suppuration or exudate, along with radiographic monitoring of bone levels, provides crucial information about implant health status. Professional cleaning and maintenance of attachments must be performed at appropriate intervals based on individual patient needs [45,46].

Patient-specific hygiene protocols need to be established, considering multiple factors including the type of attachment system used, the patient's manual dexterity, accessibility for cleaning, and overall oral health status. For patients with limited manual dexterity, supplementary cleaning aids or modified hygiene techniques may be necessary to maintain adequate peri-implant health [45,47].

Mechanical Complications

Studies have shown varying maintenance requirements for different attachment systems, with a systematic review by Vahidi and Pinto-Sinai demonstrating that implant-supported removable prostheses require periodic maintenance regardless of attachment type [45]. The most frequently encountered mechanical complications include loss of retention in attachment systems (often necessitating component replacement), screw loosening (requiring periodic tightening), denture base deterioration (necessitating relining or repair), and prosthetic tooth detachment (requiring rebonding or replacement) [44,45].

A study by Patodia et al. found that attachment-related complications represent the most frequent mechanical issue, accounting for approximately 63% of all maintenance requirements [48]. Within these attachment complications, the most common need is the replacement of nylon inserts due to wear and loss of retention, followed by replacement of the patrix (metal housing) and, less frequently, replacement of the matrix (abutment). Following attachment-related issues, denture relining represents the second most common maintenance requirement at 17% of cases. Understanding this pattern of component wear and the frequency of various complications helps clinicians develop appropriate maintenance protocols and patient education strategies [46,48].

Attachment System Considerations

Different attachment systems show varying maintenance requirements and clinical considerations. Bar attachments require monitoring for clip wear and adjustment, need attention to hygiene around the bar structure, and may experience complications related to bar joint integrity, generally showing higher initial stability but requiring more complex maintenance procedures [45,47]. Individual attachments such as Locator, OT Equator, and ball systems need regular evaluation of retention elements and typically require more frequent matrix replacement, though they show easier hygiene access and demonstrate simpler maintenance procedures overall despite potentially needing more frequent interventions [44,45].

Maintenance Protocol

A well-structured maintenance protocol should be implemented immediately following prosthetic delivery. During the first year, patients should be scheduled for recall appointments every three to four months to evaluate attachment system stability, assess prosthetic fit, and verify proper hygiene techniques. After the initial year, recall intervals may be extended to six months for cases showing stable clinical parameters. Annual radiographic examinations should be performed to monitor bone levels and implant status [44,45,48]. 

## Conclusions

ISODs represent a predictable and evidence-based solution for the rehabilitation of edentulous patients, effectively overcoming many limitations of conventional CDs while remaining cost-effective. Strong clinical evidence supports the use of two implants in the mandible, as established by the McGill consensus, providing high success rates and predictable outcomes. In the maxilla, anatomical and bone quality considerations generally necessitate the use of at least four implants to ensure adequate support and stability.

The evolution of attachment systems, particularly compact designs like the Locator, Locator R-Tx, and OT Equator systems, has significantly expanded treatment options for patients with limited inter-arch space and implant angulation challenges. Furthermore, the future of overdenture therapy is being shaped by emerging digital workflows and additive manufacturing. The integration of 3D-printed attachments and the development of high-performance polymers, such as PolyEtherEtherKetone (PEEK), offer the potential for enhanced precision and refined fabrication processes. When combined with appropriate implant placement, careful attachment selection, and systematic maintenance protocols, ISODs deliver reliable functional and quality-of-life benefits, solidifying their role as an integral component of contemporary evidence-based care for edentulous patients.
